# Transcutaneous Immunization System Using a Hydrotropic Formulation Induces a Potent Antigen-Specific Antibody Response

**DOI:** 10.1371/journal.pone.0047980

**Published:** 2012-10-24

**Authors:** Tomoka Takatani-Nakase, Erika Tokuyama, Megumi Komai, Koichi Takahashi

**Affiliations:** Department of Pharmaceutics, School of Pharmacy and Pharmaceutical Sciences, Mukogawa Women’s University, Koshien Kyuban-cho, Nishinomiya, Hyogo, Japan; Federal University of São Paulo, Brazil

## Abstract

**Background:**

Transcutaneous immunization (TCI) is a novel vaccination strategy, which is expected to have therapeutic applications. However, to develop effective TCI systems, a simple, non-invasive and safe transdermal formulation is required. This study developed a novel TCI system utilizing the co-administration of a liposoluble absorption enhancer, propylene glycol monocaprylate (PGMC) and hydrosoluble protein antigen without pretreatment of any typical adjuvants and disruption of the skin. Novel transdermal formulations were also prepared with sodium salicylate (NaSal) as a hydrotropic agent to improve the solubility of poorly water-soluble substances.

**Methodology/Principal Findings:**

The TCI system, which used a transdermal formulation containing hen lysozyme (HEL) and PGMC, solubilized with NaSal, resulted in a substantial HEL-specific antibody response in an HEL dose-dependent manner even in the absence of potent adjuvants, such as cholera toxin (CT). We also investigated whether NaSal activates antigen-presenting cells *in vitro* to clarify the mechanisms of antibody production by the hydrotropic formulation. NaSal enhanced the expression of MHC class II molecules and increased the production of IL-12 and TNF-α in dendritic cells, which were stimulated by lipopolysaccharide *in vitro*, indicating that NaSal had an effective adjuvant-like property. Moreover, the use of NaSal in the TCI system did not induce an HEL-specific, IgE-dependent anaphylactic reaction.

**Conclusion/Significance:**

Our TCI system using a hydrotropic formulation effectively and safely induced the intended immune response, and this system thus represents a new advantageous method that will result in improved TCI strategies.

## Introduction

The skin is a potential site for immunotherapy, because it has important immunological functions. For example, skin-resident antigen-presenting cells, such as Langerhans cells and dermal dendritic cells, communicate with keratinocytes, mast cells, and subsets of T lymphocytes [1]–[3]. Transcutaneous vaccination that utilizes immune responses in the skin should be a promising needle-free technique; however, administration methods, including the efficacious use of adjuvants, to attain effective transcutaneous immunization (TCI) are under development. Currently, successful immunoadjuvants for vaccines include mineral salts, immunostimulatory cytokines, liposomes, CpG oligodeoxynucleotides, microbial products, and bacterial toxins (*e.g.* cholera toxin (CT) and *Escherichia coli* heat-labile toxin) [1], [4]–[10]. These immunoadjuvants are immunologically effective; however, most are too toxic for clinical use [6]–[8].

To optimize methods for enhancing the transport of vaccine antigens, it is necessary to overcome the physical barrier of the skin surface between the body and the surrounding environment. Although techniques have been reported to remove the uppermost layer of the skin, the stratum corneum, in order to deliver antigen [11], [12],these techniques must be further improved because of serious skin damage. Moreover, a lot of interests have currently been focused on the investigation of micro/nano-meter TCI system [13], such as liposomes [4], [14], [15], patches [16] and nanoparticles [17], caused wide attention for the formulation of transcutaneous vaccines, because of their enhancements of transcutaneous delivery, the target to antigen-presenting cells, and the protection of antigen from degradation. However, the development of novel nanoscale systems for TCI is limited by the low efficiency in eliciting robust immune response. On the other hand, fatty acids, alcohols, propylene glycol, amines, and amides all enhance chemical absorption through the skin and have been used for transdermal therapeutic systems [18]–[20]. Therefore, these chemical absorption enhancers are being exploited to enhance antigen penetration through the skin barrier in TCI systems [21]. However, because of the low miscibility of the chemical absorption enhancers with water, their co-administration with the antigen is impossible [21]. Consequently, it is quite difficult to prepare transdermal formulations that consist of typically liposoluble absorption enhancers and hydrosoluble protein antigens in TCI systems.

Previously, we developed transdermal formulations using the hydrotropic phenomenon without pretreatment or disruption of the skin [22]. Hydrotropy refers to increasing the solubility of poorly water-soluble drugs by the addition of hydrotropic salts, such as urea, caffeine, nicotinamide, sodium benzoate (NaBen), and sodium salicylate (NaSal) [23]–[26]. We have found that polyol fatty acid esters (POFE) act as absorption enhancers and enable solubilization in water in the presence of hydrotropic agents, which is caused by a change in the interaction between water molecules and the aggregation of hydrotropic salts with POFE [22]. Moreover, we also reported that the hydrotropic formulation of propylene glycol monocaprylate (PGMC), a monoester of polyol fatty acid, and 5-fluorouracil (5-FU), which is an example of a hydrosoluble substance, significantly enhanced the skin penetration of 5-FU as compared with other formulations (*e*.*g*., propylene glycol and ethanol). These results suggest that the hydrotropic formulation of PGMC is potentially useful for transdermal administration of water-soluble drugs [22]. This prompted us to further use a hydrotropic formulation of PGMC in the development of effective TCI systems.

In this study, we designed a hydrotropic formulation to co-administer hen-egg lysozyme (HEL) as a model antigen and PGMC, and investigated the characteristics of the immune response against HEL with the hydrotropic formulation. Our findings show that the hydrotropic formulation with NaSal enhanced anti-HEL antibody production without a HEL-specific IgE-dependent anaphylactic reaction. In addition, NaSal acted as an adjuvant in the induction of HEL-specific immune responses.

## Materials and Methods

### Mice

BALB/c mice (6 weeks of age, female) were purchased from Japan SLC Inc. (Shizuoka, Japan). All experiments were performed in compliance with the relevant laws and institutional guidelines, and were approved by the Animal Care Committee of Mukogawa Women’s University.

### Preparation of the Transdermal Hydrotropic Formulation

The hydrotropic agents used were NaSal (Nacalai Tesque, Kyoto, Japan) and NaBen (Wako Pure Chemical Industries, Osaka, Japan). For transdermal co-administration of the hydrosoluble antigen HEL (Wako Pure Chemical Industries) and the liposoluble absorption enhancer PGMC (Nihon Surfactant Kogyo K. K., Tokyo, Japan), PGMC was dissolved in the HEL solution with the addition of NaSal or NaBen slowly with shaking, and was left for 20–40 min at room temperature until a clear solution resulted. Subsequently, 100 µL of this formulation, containing 30% (w/v) NaSal or 58% (w/v) NaBen, 1% (w/v) HEL, and 5% (v/v) PGMC, was administered to the skin of mice.

### Transcutaneous Immunization

Mice were anesthetized with an intraperitoneal injection of ethyl carbamate (1.4 g/kg, Sigma, St. Louis, MO, USA), and immunized with the transdermal hydrotropic formulation on shaved abdominal skin over an area of approximately 2.3 cm^2^, which was left for 3 h. The transdermal formulation of HEL (1.0 mg/mouse) with CT (0.1 mg/mouse) as an adjuvant was used as an experimental control for transcutaneous administration. After administration, the abdominal skin was swabbed gently with distilled water. Two and 4 weeks after the primary immunization, the formulation was again administered to the mice in the same manner as that for the primary immunization. For measurements of serum antibodies, blood samples were taken from the eyes every 2 weeks. Abdominal skin samples were collected 12 weeks after primary immunization and homogenized in Tris-buffered saline (TBS). The skin homogenate was centrifuged (14,000 × *g*, 5 min, 4°C) to remove insoluble material, and the resulting supernatant was used for the determination of antibodies in the skin.

### Evaluation of Effect of NaSal Dose on Transcutaneous Immunization

**Figure 1:**
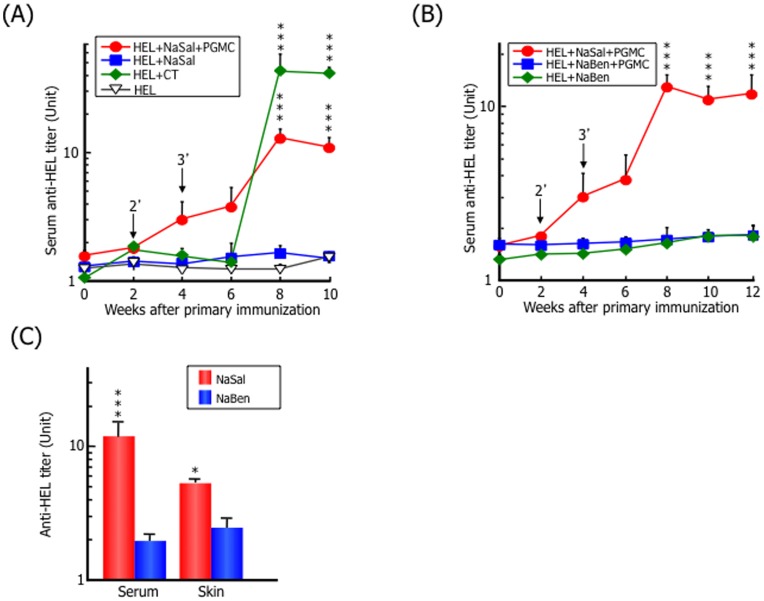
High levels of anti-HEL antibody production after TCI using the hydrotropic formulation solubilized with NaSal. (A) The transdermal formulations of HEL (1.0 mg/mouse) alone (▾) or together with (•) or without (▪) 5% PGMC as an absorption enhancer in the presence of 30% NaSal as a hydrotropic agent, or HEL (1.0 mg/mouse) in CT as an adjuvant (♦) were applied directly to the shaved abdominal skin of BALB/c mice for 3 h as described in the Materials and Methods. Immunizations for each group of mice were performed in the same manner at 0, 2, and 4 weeks. Anti-HEL antibody titers in serum samples were determined every 2 weeks by ELISA. ^***^, *p*<0.0001, compared with HEL without adjuvant. (B) Effects of the hydrotropic agent for transdermal immunization was tested by replacing 30% NaSal with 58% NaBen in the formulation of HEL (1.0 mg/mouse) with (▪) or without (♦) 5% PGMC. ^***^, *p*<0.0001, compared with HEL without adjuvant. Arrowhead indicates transdermal immunization point. (C) Anti-HEL antibody titers in serum and skin were determined at 12 weeks after the primary immunization with the transdermal formulation of HEL (1.0 mg/mouse) and 5% PGMC in the presence of 30% NaSal or 58% NaBen. ^*^, *p*<0.05; ^***^, *p*<0.0001, compared with HEL and PGMC in the presence of NaBen. The data represent the mean and SD of six mice in each experimental group.

It is required >30% NaSal to dissolve PGMC in the HEL solution [22]. To determine effect of low dose NaSal on TCI, PGMC was completely dissolved in the HEL solution with the addition of other hydrotropic agent, NaBen, which does not induce immune response in <$>\raster="<$>≦ 58% (w/v) of NaBen as shown in Fig. 1C. The 100 µL of hydrotropic formation, containing 1% (w/v) HEL and 5% (v/v) PGMC solubilized with 20% (w/v) NaSal and 39% (w/v) NaBen, 10% (w/v) NaSal and 42.4% (w/v) NaBen, 4% (w/v) NaSal and 45.7% (w/v) NaBen, or 58% (w/v) NaBen without NaSal was immunized to the skin of mice at 0, 2 and 4 weeks as described above *Transcutaneous immunization*.

### Enzyme-linked Immunosorbent Assay (ELISA)

The serum and skin levels of HEL-specific immunoglobulin were measured using an ELISA and standardized against a positive control antiserum obtained by hyperimmunization of mice with HEL in FCA. The hyper-immunization schedule involved primary and secondary immunization, separated by 2 weeks, of subcutaneous injection of HEL in FCA (25 µg/mouse; Difco, Detroit, MI, USA) and the serum sample was taken from eyes 4 weeks after the second immunization. The ELISA was performed as follows: a 96-well plate (Sumitomo Bakelite, Tokyo, Japan) was coated with 5 µg/mL HEL in 50 mM carbonate buffer (pH 9.6) overnight at 4°C. After three washes with TBS-T as the wash buffer (TBS containing 0.05% Tween-20), each well was blocked with TBS containing 3% bovine serum albumin (BSA; Sigma) at room temperature for 1.5 h to block non-specific binding. After washing with TBS-T, serum samples diluted with TBS (1∶2000) in triplicate were added to the plates, and then incubated overnight at 4°C. The plates were washed with TBS-T, and alkaline phosphatase-conjugated antibody to mouse polyvalent immunoglobulins (Sigma) or mouse IgG, IgA, and IgM (Sigma) were added at room temperature for 2 h. After washing with TBS-T, the reaction was developed with p-nitrophenyl phosphate (Sigma) for 20 min and the absorbances at 405 nm were read. The results were expressed as antibody units calculated from the standard curve obtained from the hyperimmune mouse serum. For the standard curve, the highest dilution resulting in a value of serum above the negative control is defined as 1 unit, the unit of standard serum is assigned according to dilution rate, and plotted absorbances on the ordinate against log scale of standard serum unit. Negative control is an experiment without any sample on each experimental plate to monitor nonspecific reactions.

### Generation of Mouse Bone Marrow-derived Dendritic Cells (BMDCs)

BMDCs were generated *in vitro* using procedures reported previously [27], [28]. Briefly, bone marrow cells were isolated from BALB/c mice (8 weeks of age, female) and cultured in bacteriological Petri dishes (2×10^6^/90-mm dish) in RPMI 1640 medium (Invitrogen, Carlsbad, CA, USA), supplemented with 100 U/mL penicillin (Nacalai Tesque), 100 µg/mL streptomycin (Nacalai Tesque), 10% fetal calf serum (FCS; Invitrogen), 250 U/mL mouse granulocyte macrophage-colony stimulating factor (PeproTech, Rocky Hill, NJ, USA), and 50 µM 2-mercaptoethanol (Sigma) for 10 days. Non-adherent and loosely adherent cells were harvested and used as BMDCs (>80% CD11c^+^). BMDCs were cultured in 48-well culture plates at 2.5×10^5^ cells/500 µL and stimulated with 0.001 µg/mL lipopolysaccharide (LPS; Sigma) in the presence or absence of NaSal.

### Flow Cytometry Analysis

After 72 h of stimulation with LPS in the presence or absence of NaSal, single-cell suspensions were incubated with biotin-conjugated antibody to I-A^d^ (BD PharMingen, Hamburg, Germany) for 30 min on ice. The cells were washed with phosphate-buffered saline (PBS) containing 1% bovine serum albumin (BSA) and stained with R-phycoerythrin-conjugated streptavidin (Dako, Glostrup, Denmark) for 45 min on ice. After incubation, the cells were washed with 1% BSA in PBS, fixed in 2% paraformaldehyde (Nacalai Tesque), and analyzed using a FACSCalibur flow cytometer and CellQuest software (BD Biosciences, Mountain View, CA, USA).

### Cytokine Measurement by ELISA

After 72 h of stimulation with LPS in the presence or absence of NaSal, the amounts of interleukin (IL)-12 p40/p70 and tumor necrosis factor (TNF)-α in the culture supernatants were determined using mouse ELISA kits (Invitrogen).

### Detection of IgE-dependent Anaphylactic Reactions by the Abdominal Wall (AW) Method

The shaved abdominal skin of BALB/c mice was immunized with a transdermal formulation of HEL (1.0 mg/mouse) alone or co-administered with HEL (1.0 mg/mouse) and 5% PGMC, solubilized in the presence of 30% NaSal for 3 h. Immunizations for each group of mice were performed in the same manner at 0, 2, and 4 weeks. Six weeks after the primary immunization with the transdermal formulation, mice were sensitized intraperitoneally with HEL (2.5 µg/mouse) in FIA. After 9 days of sensitization, an IgE-dependent anaphylactic reaction was detected by the AW method, as described previously [29], [30]. Briefly, mice were injected with 0.1 mL of 1% Evans blue dye intravenously, anesthetized with ether, and the abdominal wall was exposed without damage. Five minutes after injecting the dye, HEL (5 µg/50 µL/site) was administered into the abdominal wall. Seven minutes after treatment, the mice were sacrificed and the abdominal wall was removed. The mean of the long and short diameters of the blue area was evaluated on the abdominal wall and assigned a vascular permeability value (VPV), from 0 to 100, with scores representing the following: VPV = 0, *x*<1.5; VPV = 25, 1.5 ≦ *x*<4.5; VPV = 50, 4.5 ≦ *x*<7.5; VPV = 75, 7.5 ≦ *x*<12.5; and VPV = 100, 12.5 ≦ *x*; where *x* indicates the mean of the diameters (mm).

### Statistical Analysis

All statistical analyses were performed using GraphPad Prism (ver. 5.00; GraphPad, San Diego, CA, USA). Statistical differences among the groups with respect to anti-HEL titers, cell surface marker expression levels, and cytokines were analyzed using one- or two-way analysis of variance (ANOVA) followed by Dunnett’s test or Bonferroni multiple comparison tests. Statistical significance among the groups with respect to the anaphylactic score was analyzed using the Kruskal-Wallis test followed by Dunn’s multiple comparison test. Differences were considered significant when the calculated *p*-value was <0.05.

## Results

### High Level of Anti-HEL Antibody Production in Mice Immunized with Co-administration of HEL and PGMC Solubilized in the Presence of NaSal

To examine the efficacy of TCI for HEL using the transdermal hydrotropic formulation, BALB/c mice were immunized with HEL using CT as adjuvants, or PGMC in the presence of NaSal as a hydrotropic agent without any adjuvants, and serum anti-HEL antibody titers were monitored. The HEL and PGMC are hydrophilic and hydrophobic, respectively, and the combination is very difficult to prepare, because of their low solubility. On the other hand, we successfully prepared the dissolved mixture of HEL and PGMC in the presence of NaSal. Co-administration of HEL and PGMC solubilized in the presence of NaSal as a transdermal hydrotropic formulation resulted in substantial HEL-specific antibody production, even in the absence of a potent adjuvant, the higher anti-HEL antibody production was observed in the co-administration of HEL, PGMC, and NaSal till six weeks passed than that of HEL and CT with significant differences, and delayed response was observed in the administration of HEL and CT at eight weeks (Fig. 1A). By contrast, the administration of HEL alone or HEL in the presence of NaSal without PGMC did not induce a significant antibody response, suggesting that PGMC is needed for the transdermal absorption of HEL, and NaSal may play an important adjuvant-like role in the immune response. We also observed the localization of FITC-labeled HEL in co-administration with PGMC and NaSal to the skin using fluorescent microscopy. The HEL, which was co-administrated with PGMC and NaSal, localized in the stratum corneum after 3 hours of the administration (Fig. S1). On the other hand, it was quite difficult to observe the localization of the HEL without PGMC and NaSal in the stratum corneum (Fig. S1).

To investigate whether other hydrotropic agents induced anti-HEL antibody production, we compared immunization in the presence of NaSal or NaBen (Fig. 1B). Co-administration of HEL and PGMC in the presence of NaBen did not induce a significant antibody response, demonstrating that hydrotropic agents cause solubilization of PGMC in water, but the immunoenhancing effect is apparently specific to NaSal.

**Figure 2:**
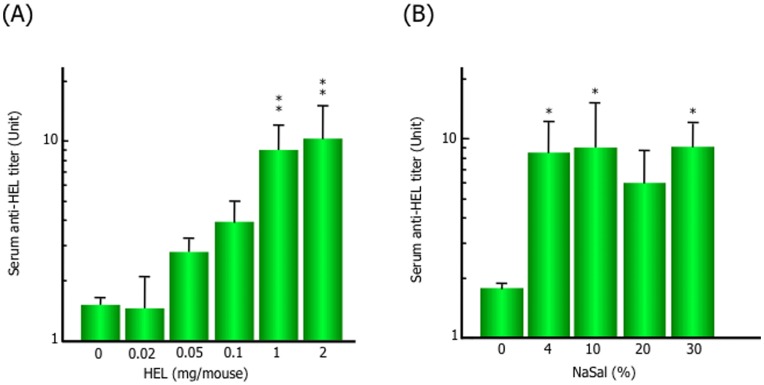
Evaluation of dose-dependent effects of HEL and NaSal on immune response after TCI. The shaved abdominal skin of BALB/c mice was immunized with the transdermal formulation containing the indicated dose of HEL from 0 to 2.0 mg and 5% PGMC in the presence of 30% NaSal (A) or HEL (1.0 mg/mouse) and 5% PGMC with the indicated dose of NaSal as described in the Materials and Methods (B) for 3 h. Immunizations for each group of mice were performed in the same manner at 0, 2, and 4 weeks. Anti-HEL antibody titers in serum were determined at 10 weeks after the primary immunization. Data represent the mean and SD of six mice in each experimental group. ^**^, *p*<0.001, compared with the control group without HEL treatment. ^*^, *p*<0.05, compared with the control group without NaSal treatment.

**Figure 3:**
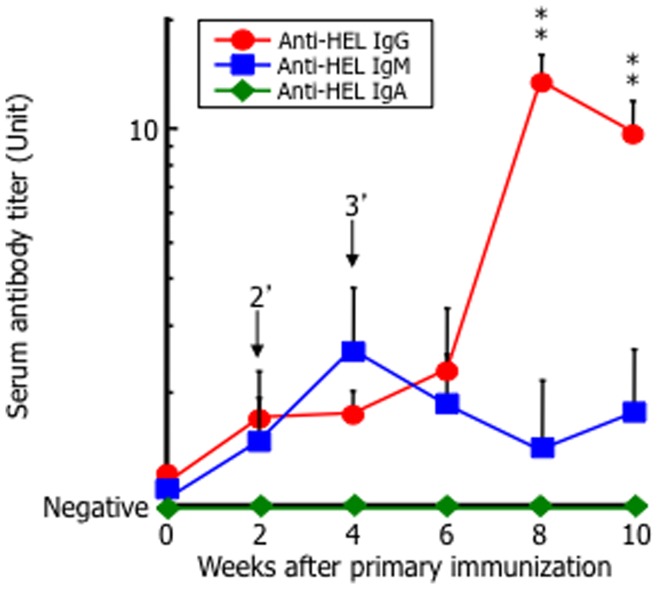
Analysis of HEL-specific antibody subtypes. The shaved abdominal skin of BALB/c mice was immunized with a transdermal hydrotropic formulation containing HEL (1.0 mg/mouse) and 5% PGMC with 30% NaSal for 3 h. Immunizations for each group of mice were performed in the same manner at 0, 2, and 4 weeks. Serum samples collected from the mice every 2 weeks were assayed for HEL-specific antibody subtype (IgG, IgM, IgA) titers by ELISA. The data represent the mean and SD of six mice in each experimental group. ^**^, *p*<0.001, compared with the primary immunization at week 0. Arrowhead indicates transdermal immunization point.

Twelve weeks after the primary immunization, anti-HEL antibody production in the abdominal skin increased in the mice administered HEL and PGMC with NaSal, indicating that immunization in the presence of NaSal efficiently induced the intended immune response (Fig. 1C). To determine the optimal conditions of our TCI system using the hydrotropic formulation, we analyzed the influence of HEL and NaSal doses. As shown in Figure 2A, the titers of serum antibodies increased in a HEL dose-dependent manner at 10 weeks after the primary immunization. Moreover, as a result of assay using hydrotropic formulation containing HEL and PGMC with different NaSal dose completely solubilized by the addition of NaBen, which was confirmed to play non-adjuvant-like role as shown in Fig. 1A, the titers of serum antibodies increased with ≧4% NaSal and the immune response was largely independent of the NaSal dose (Fig. 2B). In the case of <4% of NaSal dose, immune responses were not reproducibly shown, because of their unstable induction of immune response by low dose of NaSal treatment (data not shown). We next evaluated which antibody subtypes were induced in mice immunized with our TCI using a hydrotropic formulation. As shown in Figure 3, HEL-specific IgM antibodies tended to increase after immunization with the hydrotropic formulation and reached a peak at week 4, then decreased, whereas the level of HEL-specific IgG antibodies increased gradually in mice immunized with the hydrotropic formulation. Anti-HEL IgA antibody production was not observed in mice immunized with the hydrotropic formulation.

### NaSal has Adjuvant-like Properties and Promotes Lipopolysaccharide-stimulated Maturation of Bone Marrow Dendritic Cells in vitro

**Figure 4:**
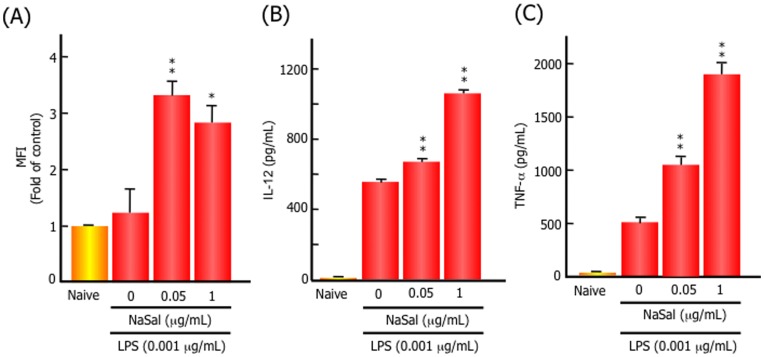
NaSal enhanced the expression of MHC class II molecules and the secretion of cytokines in LPS-stimulated BMDCs. BMDCs were differentiated from bone marrow cells in mice by the addition of mouse granulocyte macrophage-colony stimulating factor (250 U/mL) and 2-mercaptoethanol (50 µM) for 10 days. BMDCs were stimulated with LPS (0.001 ng/mL) in the presence or absence of NaSal for 72 h. (A) Cell surface expression levels of MHC class II molecules were determined by FACS. Mean fluorescence intensity (MFI) is expressed as the mean and SD of three independent experiments. (B) The concentrations of IL-12 and TNF-α in the culture supernatants were determined by ELISA. The data represent the mean and SD of three independent experiments. ^*^, *p*<0.05; ^**^, *p*<0.001, compared with the control group with LPS treatment alone.

NaSal enhanced the magnitude of antibody production *in vivo*. Therefore, we hypothesized that NaSal exerts this effect by activating antigen presentation by dendritic cells. To test this, we used mouse BMDCs stimulated with LPS, a potent inducer of innate cytokines, in the presence or absence of NaSal *in vitro*, and examined the expression of major histocompatibility complex (MHC) class II molecules and the secretion of cytokines. After LPS stimulation, NaSal significantly upregulated the expression of MHC class II molecules as compared with LPS alone (Fig. 4A). Moreover, NaSal enhanced the production of TNF-α and IL-12, depending on the dose of NaSal in culture (Fig. 4B, C). These findings demonstrated that NaSal could activate BMDCs and play a role as an adjuvant in the immune response.

### Effect of the Transdermal Vaccine with NaSal on IgE-dependent Anaphylactic Reactions

**Figure 5:**
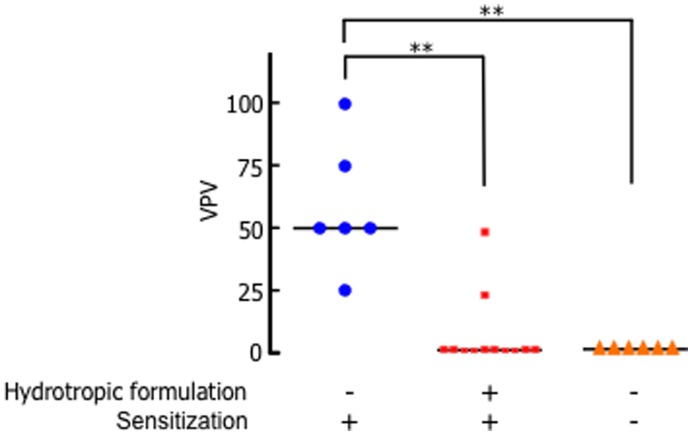
Transcutaneous immunization with NaSal did not induce an IgE-dependent anaphylactic reaction. The shaved abdominal skin of BALB/c mice was immunized with a transdermal hydrotropic formulation containing HEL (1.0 mg/mouse) and 5% PGMC solubilized in the presence of 30% NaSal for 3 h as described in the Methods. Immunizations for each group of mice were performed in the same manner at 0, 2, and 4 weeks. At 6 weeks, mice were sensitized intraperitoneally with HEL (2500 µg/mouse) in FIA. After 9 days of sensitization, the mice were treated with HEL (5 µg/site) on the abdominal wall. Individual VPVs (vascular permeability values) are displayed with the median (bars) of 6–12 mice in each experimental group. ^**^, *p*<0.001, compared with the group sensitized with HEL.

The production of IgE in transcutaneous immunization leads to symptoms of allergic disease, namely type-I hypersensitivity reactions. To investigate whether TCI for HEL using the transdermal hydrotropic formulation involved HEL-specific IgE responses, the IgE-dependent anaphylactic reaction was assessed using AW method. Six weeks after the primary immunization with HEL alone or HEL and PGMC in the presence of NaSal, mice were sensitized intraperitoneally with HEL (2500 µg/mouse) in Freund’s incomplete adjuvant (FIA). After 9 days of sensitization, no HEL-specific anaphylactic reaction was observed based on the AW method assay in mice immunized with HEL using the transdermal hydrotropic formulation, while mice immunized with HEL alone and positive control mice did show a significant anaphylactic response (Fig. 5). These data suggest that the administration of HEL using the transdermal hydrotropic formulation via the skin did not induce HEL-specific allergic responses.

## Discussion

This study demonstrated that transdermal co-administration of HEL and PGMC solubilized in the presence of NaSal resulted in a substantial HEL-specific antibody response, similar to immunization using CT as adjuvants. In addition, it markedly attenuated the HEL-specific IgE-dependent anaphylactic reaction. The use of NaSal in transdermal vaccine delivery showed effective adjuvant-like properties in the induction of HEL-specific immune responses. Therefore, we developed a novel TCI system using the hydrotropic agent NaSal.

This TCI system was dependent on the antigen dose in the presence of NaSal, and NaSal promoted the expression of MHC class II molecules and increased the production of IL-12 and TNF-α in dendritic cells, which were stimulated by LPS *in vitro*, suggesting that NaSal had an adjuvant-like effects via activation of antigen presenting cells. However, it has remained unclear how NaSal affects the immune response. We observed the localization of FITC-labeled HEL in co-administration with PGMC and NaSal to the skin. Fluorescent microscopic observation showed that FITC-HEL localized in the stratum corneum after 3 hours of the administration (Fig. S1). These results suggest that the accumulated antigen in the stratum corneum may efficiently lead to the immune response. Regarding the signal transduction to immune adjuvant activity, previous reports have shown that pertussis toxin and CT act as adjuvants, inducing the up-regulation of MHC class II molecules and enhancing the production of IL-12 by promoting clonal expansion of T cells via G protein-mediated activation of antigen presenting cells (APCs) [31], [32]. The data indicate that salicylate and other non-steroidal anti-inflammatory drugs (NSAIDs) interact with a G protein in the neutrophil plasmalemma, leading to uncoupled post-receptor signaling events [33]. It is possible that the adjuvant properties of NaSal could be the result of G protein modulation, although further studies are needed to address this. The CC chemokine receptor, CCR7 also has been described essential mediator for migration of skin dendritic cells of CD11c (+) and MHC class II (high) [34]. NaSal may be involved in trafficking the skin dendritic cells to lymph node through CCR7 because our data indicate the up-regulation of MHC class II molecules by NaSal *in vitro*. Moreover, Hsia *et al*. demonstrated that the oral administration of acetylsalicylic acid before influenza vaccination significantly increased the immune response in a placebo-controlled, double-blind, randomized trial [35]. Taken together with our data, these findings suggest that analysis of the immune adjuvant-like mechanisms of NaSal can provide novel strategies for transdermal vaccine delivery.

Although the adjuvants currently approved for clinical use are only aluminum-containing compounds, such as hydroxides and phosphates (alum), alum is known to stimulate only humoral responses and is known to induce IgE antibody production, which can lead to an allergic response to the vaccine [6], [36]. Our data showed that immunization with HEL and PGMC in the presence of NaSal through the skin did not induce an antigen-specific IgE-dependent anaphylactic reaction, suggesting the potential use of NaSal in the development of a vaccine with a low or no allergic reaction and for its use in immunotherapy.

Our TCI system showed the delay immune response and booster effect similarity to recent findings about TCI system by patches [16], suggesting the memorization of the response might possibly be induced. In the skin epidermis, effector/memory T cells, principally cytotoxic CD8 T cells contribute to the antigen specific effector/memory cellular responses [37], [38], however, the detail of the immune memory in TCI system remains unclear, and further studies are needed to understand the mechanisms and to improve more effect TCI system allowing rapidly immune response.

Our findings indicate that the TCI system described here, which used a hydrotropic formulation with NaSal, induced antigen-specific immune responses and IgE-selective unresponsiveness. This is the first reported demonstration of the potential use of NaSal as a hydrotropic agent and an immune adjuvant in the development of a TCI system. Overall, a TCI system using a hydrotropic formulation may offer a new strategy for the development of a safe and effective method for transdermal vaccine delivery.

## Supporting Information

Figure S1 Localization of FITC-labeled HEL in mouse skin after TCI with NaSal treatment. The shaved abdominal skin of BALB/c mice was immunized with a transdermal hydrotropic formulation containing FITC-HEL (1.0 mg/mouse) alone or together with 5% PGMC solubilized in the presence of 30% NaSal. After 3 h, the abdominal skin was swabbed gently, and the localization of FITC-labeled HEL in the skin was observed using fluorescent microscopy. The fluorescence images were captured from application sites after treatment using the Nikon fluorescent microscopy system (Tokyo, Japan).
(TIFF)
