# Supplementary figures and images for: Transcutaneous Immunization System Using a Hydrotropic Formulation Induces a Potent Antigen-Specific Antibody Response
Source: PLoS One. 2012 Oct 24;7(10):e47980. doi: 10.1371/journal.pone.0047980 (PMC3480500; doi:10.1371/journal.pone.0047980)

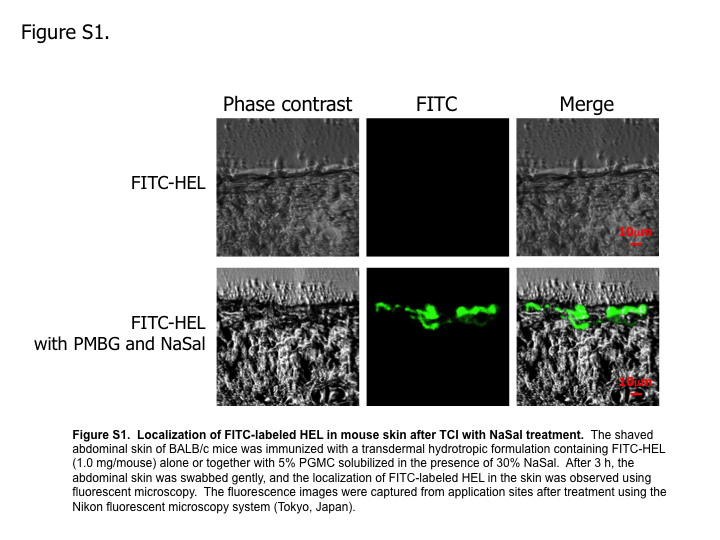

Supplement: Figure S1 — Localization of FITC-labeled HEL in mouse skin after TCI with NaSal treatment. The shaved abdominal skin of BALB/c mice was immunized with a transdermal hydrotropic formulation containing FITC-HEL (1.0 mg/mouse) alone or together with 5% PGMC solubilized in the presence of 30% NaSal. After 3 h, the abdominal skin was swabbed gently, and the localization of FITC-labeled HEL in the skin was observed using fluorescent microscopy. The fluorescence images were captured from application sites after treatment using the Nikon fluorescent microscopy system (Tokyo, Japan). (TIFF) [file pone.0047980.s001.tif]
